# Spin-Resolved Magneto-Tunneling and Giant Anisotropic *g*-Factor in Broken Gap InAs-GaSb Core–Shell Nanowires

**DOI:** 10.1021/acs.nanolett.3c02559

**Published:** 2024-01-08

**Authors:** Vito Clericò, Pawel Wójcik, Andrea Vezzosi, Mirko Rocci, Valeria Demontis, Valentina Zannier, Álvaro Díaz-Fernández, Elena Díaz, Vittorio Bellani, Francisco Domínguez-Adame, Enrique Diez, Lucia Sorba, Andrea Bertoni, Guido Goldoni, Francesco Rossella

**Affiliations:** † Nanolab-Nanotechnology Group, Departamento de Física Fundamental, Universidad de Salamanca, Plaza de la Merced, s/n., 37008-Salamanca, Spain; ‡ AGH University of Krakow, Faculty of Physics and Applied Computer Science, Al. Mickiewicza 30, 30-059 Krakow, Poland; § Dipartimento di Scienze Fisiche, Informatiche e Matematiche, Università di Modena e Reggio Emilia, Via Campi 213/a, I-41125 Modena, Italy; ∥ NEST, Scuola Normale Superiore e Istituto di Nanoscienze-CNR, Piazza san Silvestro 12, I-56127 Pisa, Italy; ⊥ Department of Physics, University of Cagliari, S.P. Monserrato-Sestu, Monserrato, 09042, Italy; # GISC, Departamento de Física de Materiales, Universidad Complutense de Madrid, Avenida Complutense, s/n, Ciudad Universitaria, 28040 Madrid, Spain; ○ Dipartimento di Fisica, Università di Pavia, Via Agostino Bassi, 6, 27100 Pavia, Italy; □ S3, Istituto Nanoscienze-CNR, Via Campi 213/a, I-41125 Modena, Italy

**Keywords:** high magnetic field, spin-resolved transport, magneto-tunneling, *g*-factor, broken gap, InAs-GaSb, core−shell nanowires

## Abstract

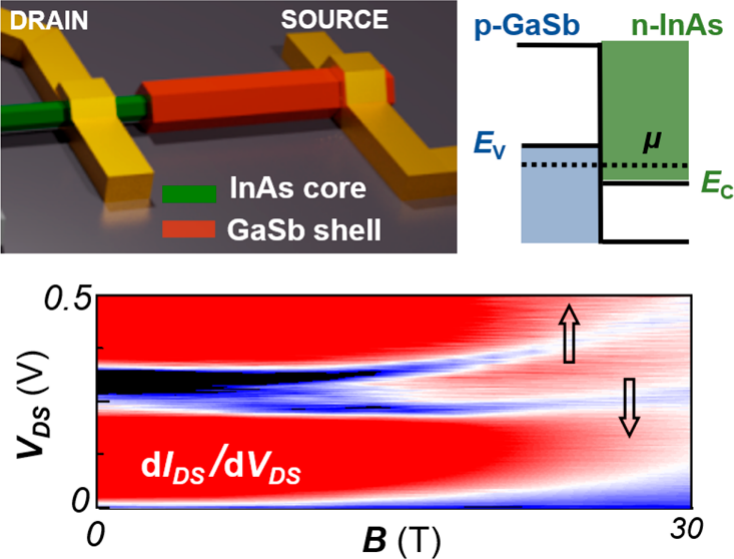

We experimentally and computationally investigate the magneto-conductance across the radial heterojunction of InAs-GaSb core–shell nanowires under a magnetic field, *B*, up to 30 T and at temperatures in the range 4.2–200 K. The observed double-peak negative differential conductance markedly blue-shifts with increasing *B*. The doublet accounts for spin-polarized currents through the Zeeman split channels of the InAs (GaSb) conduction (valence) band and exhibits strong anisotropy with respect to *B* orientation and marked temperature dependence. Envelope function approximation and a semiclassical (WKB) approach allow to compute the magnetic quantum states of InAs and GaSb sections of the nanowire and to estimate the *B*-dependent tunneling current across the broken-gap interface. Disentangling different magneto-transport channels and a thermally activated valence-to-valence band transport current, we extract the *g*-factor from the spin-up and spin-down d*I*/d*V* branch dispersion, revealing a giant, strongly anisotropic *g*-factor in excess of 60 (100) for the radial (tilted) field configurations.

Tunable electron spin coherence, a key requirement for spintronics and spin-based quantum computation,^1−3^ can be achieved in a material where spins are robust against relaxation mechanisms as well as sizably coupled to carrier orbital motion.^4^ Unfortunately, these two desirable requirements are conflicting in most semiconductor materials, and an impressive effort of material and device engineering is being carried out in order to realize artificial novel systems enabling, for instance, the control of spin coherence via an external field.^5,3^ In this context, semiconductor nanowires (NWs) and NW heterostructures have gained a prominent role as platforms to demonstrate quantum supremacy.^6^ As an example, they boosted the search for the elusive Majorana Fermions,^7,8^ thanks to their unique material properties and topologies and to the possibility to engineer very high^9,10^ or gate-controllable^11^ Landé *g*-factors. Noticeably, in contrast with common thought and general expectation that quantum-confinement reduces the *g*-factor, a very large electronic *g*-factor has been reported in recent years for small effective mass semiconductor NWs investigated in low-temperature magneto-transport experiments.^9,12^ From the theoretical point of view, a large *g*-factor enhancement in nanoscale semiconductors, including nanowires and quantum dots, has been predicted and ascribed to orbital contributions to the L-S coupling.^13^

In this Letter we report magneto-transport measurements in InAs-GaSb core–shell (C–S) NW-based devices under a magnetic field up to 30 T and in the temperature range 4.2–200 K. We investigate the interband tunneling across the radial broken-gap heterojunction embedded in the C–S NW by monitoring the dependence of the differential conductivity upon a magnetic field *B* applied radially or tilted with respect to the NW axis. A negative differential resistance (NDR) feature, characteristic of the Esaki effect arising from the broken-gap band alignment,^14,15^ splits into a doublet at finite *B*, which is attributed to spin-polarized currents arising from the Zeeman splitting of the InAs conduction band and GaSb valence band. The field evolution of the two spin-resolved NDR branches reflects the interplay between an almost-quadratic blue-shift, accounting for the increasing magnetoresistance of the InAs core, and a linear red-shift, due to the effective narrowing of the broken gap. Using the envelope function approximation and the WKB semiclassical approach to calculate the tunneling current across the broken-gap interface, we computationally reproduce the experimental differential magneto-conductance, and we unambiguously assign the spin-filtered channels, extracting the corresponding *g*-factor. A large, highly anisotropic *g*-factor in the range 60–100 is estimated. A temperature-induced suppression of the spin-down polarized NDR feature is observed, which can be accounted for by invoking a spin-independent thermally activated valence-to-valence band current. This shows that NDR spin filtering is robust against thermal excitations due to the very large *g*-factor. Our results, concerning a nanoscale heterostructure, where electrons and holes coexist in adjacent layers, may foster fundamental studies on low-dimensional Coulomb drag and point out new routes to explore exotic topological phases of matter due to carrier interaction.^16^ At the same time, in the presence of temperature gradients across the individual nanostructures, our work may open new perspectives for the study of spin thermoelectric conversion and harvesting at the nanoscale,^17−19^ as well as for spintronic technologies at large.

The InAs-GaSb C–S NWs investigated in this study were grown by catalyst-free chemical beam epitaxy (CBE) on Si(111) substrates.^20^ The core consists of a nominally undoped InAs NW, with length *L* ≈ 2 μm and diameter *d* ≈ 60 nm. The InAs NW core is covered by a ≈30 nm thick GaSb shell. The structural properties and chemical composition of the nanostructure are reported elsewhere.^14^ The NWs were mechanically detached from the growth substrate and transferred by dropcasting to a p^+2^Si/SiO_2_ substrate. Figure 1a shows a schematic view of the NW-based device architecture developed in this work, with circuital elements and applied magnetic field orientations depicted in overlay. An in-plane magnetic field up to 30 T can be applied either radially with respect to the NW or tilted at 45° with respect to the NW axis, i.e., combining radial and axial components. In fact, the device chip is mounted onto a rotating stage with 0.1° angular uncertainty and equipped with a calibrated Hall probe, which measures the out-of-plane component of the magnetic field. The in-plane configuration is achieved by rotating the stage until the Hall probe signal reaches its minimum. Figure 1b shows a false-colors scanning electron microscopy (SEM) micrograph of one of the fabricated C–S NW-based devices. A two-step electron beam lithography together with a highly selective GaSb etching protocol were employed to define Ti/Au (10/140 nm) electrical contacts on both the InAs core of the nanostructure and the GaSb shell.^14,21,22^ A finite source-drain voltage bias (*V*_DS_) applied using the yellow-colored electrodes in Figure 1a,b induces an electrical current (*I*_DS_) across the InAs-GaSb radial heterojunction. The *k*·*p* calculations (see Supporting Information, Section 1) indicate that, due to the large diameter of the InAs core and thickness of the GaSb shell and the resulting small confinement energies, strongly band-inverted metallic features occur between the GaSb valence band edge (*E*_v_) and the InAs conduction band edge (*E*_c_) at the C–S interface at zero bias. At zero or vanishingly small magnetic field, the broken-gap band alignment occurring at the heterojuntion (see Figure 1c) enables tunneling processes analogous to those promoting the Esaki effect, resulting in a marked nonlinearity of *I*_DS_ as a function of *V*_DS_, including a NDR region.^14,21^Figure 1d pictorially shows the impact of an externally applied magnetic field, *B*, on the band edges in the two semiconductors of our heterostructure. On the one hand, the band edges *E*_v_ and *E*_c_ shift with *B* with parabolic dependence, with opposite orbital evolutions due to opposite sign of the effective mass. For sufficiently small *B*, Δ*E* = *E*_v_ – *E*_c_ remains positive, preserving the broken-gap condition at zero bias. On the other hand, the bands are subjected to linear Zeeman splitting, whose magnitude is controlled by the carrier effective *g*-factor in the core and shell regions of the NW. Therefore, as sketched in Figure 1e, separate broken-gap conditions occur for the two spin populations, each carrying a highly (oppositely) spin-polarized tunneling currents. Ultimately, this is envisioned to force the NDR region to split into two spin-polarized components. The expected qualitative behavior of *I*_DS_ (*V*_DS_) in our C–S NW at zero magnetic field and at finite radial magnetic field is pictorially depicted in Figure 2a and b, respectively.

**Figure 1:**
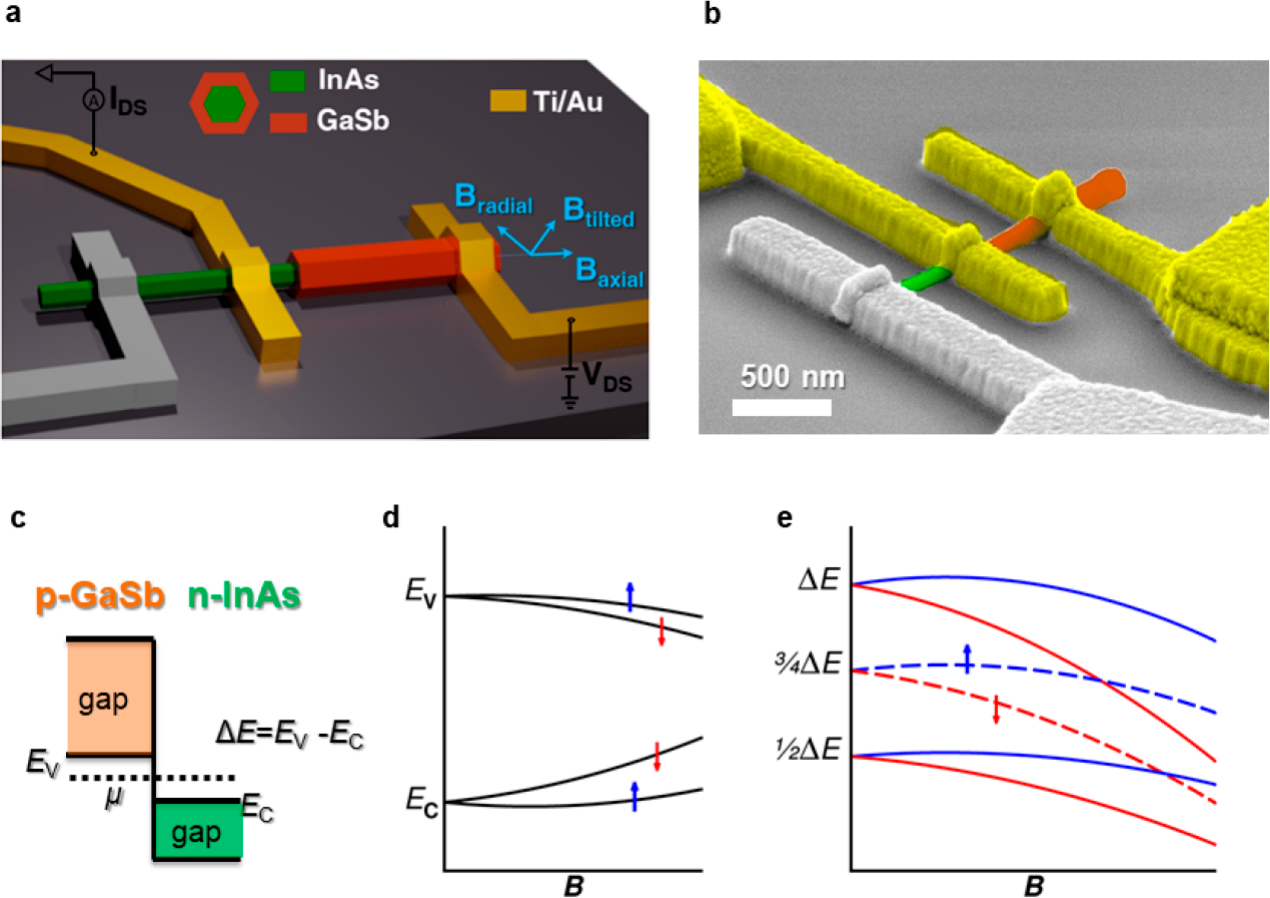
(a) Cross-sectional schematics of the InAs-GaSb C–S NW (left) and the measurement configuration (right). The magnetic field is directed along the NW radius or tilted, including an axial component. (b) SEM image of a prototypical device identical to the ones employed in the experiment (gray = SiO_2_/Si, yellow = Ti/Au, pink = GaSb, green = InAs). (c) Band alignment at the InAs-GaSb interface without applied voltage bias and magnetic field. (d) Schematic evolution of the band edges of the top valence band of GaSb and bottom conduction band of InAs in magnetic field, where arrows indicate carrier spins. (e) Evolution of the NDR condition as a function of *B*. The centers of the two NDR features, corresponding to a dip in the differential conductance, occur at a junction bias of 3/4Δ*E* and are indicated by the two dotted lines (one for each spin channel).

**Figure 2:**
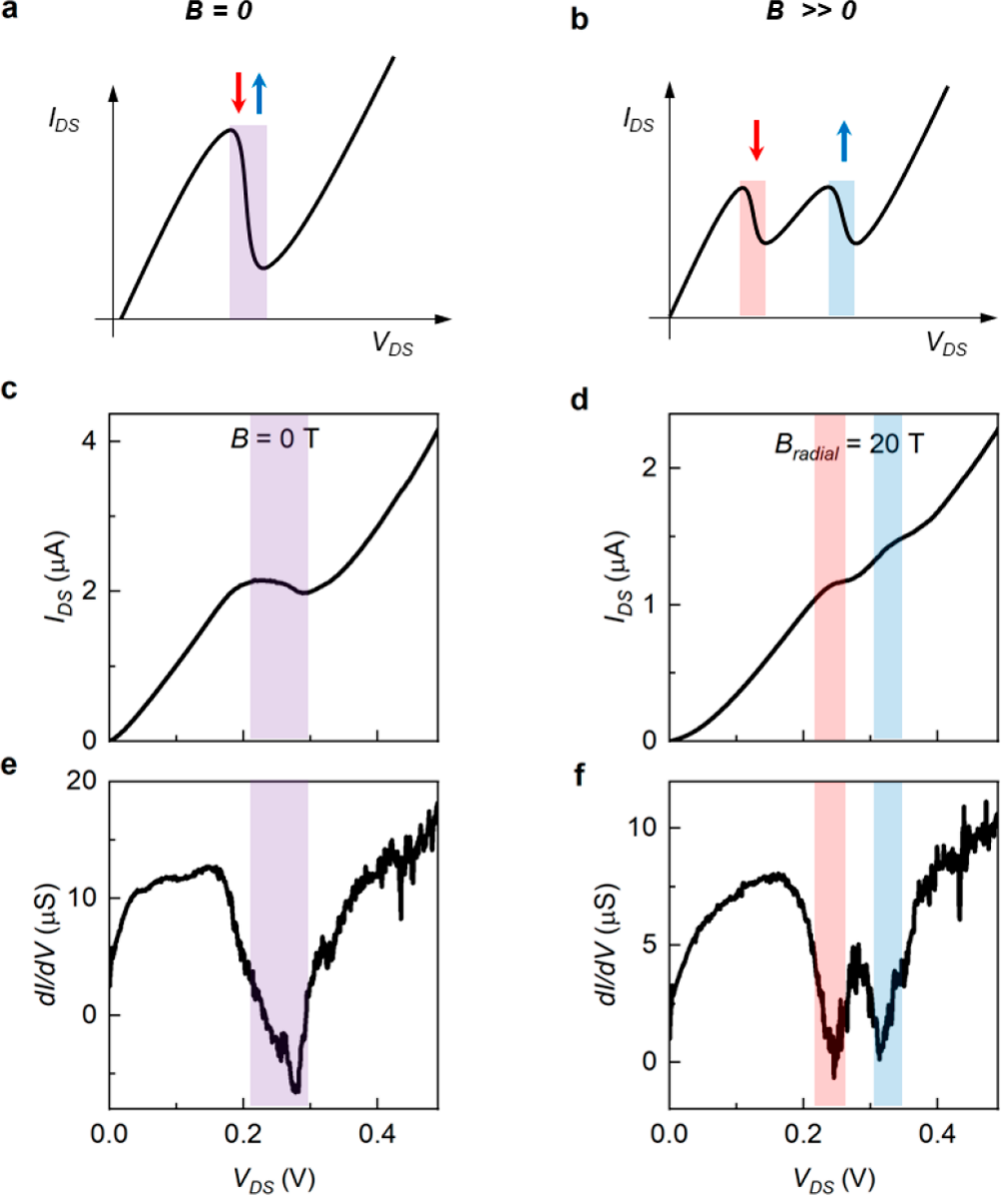
Schematic representation of the current–voltage characteristic of InAs-GaSb C–S NW device at (a) zero magnetic field and (b) finite magnetic field. The arrows label the spin-polarization of the NDR features. Measured *I*–*V* curves at *T* = 4.2 K at (c) *B* = 0 and (d) 20 T. (e, f) Differential conductance obtained from panels (c) and (d), respectively.

Figure 2c and d report experimental *I*_DS_ (*V*_DS_) curves measured in one of our devices at temperature *T* = 4.2 K and magnetic field *B* = 0 and *B* = 20 T, respectively. At *B* = 0, we observe a single NDR region, which is best resolved in the d*I*/d*V*(*V*_DS_) curve obtained by numerical derivative, as reported in Figure 2e, showing a dip at *V*_DS_ ∼ 0.28 V. The observed NDR broadening can be ascribed to the finite size of the broken gap junction and to the junction nonidealities introduced by the growth process of the nanomaterial and the fabrication process of the nanodevice (e.g., chemical or structural defect of the InAs/GaSb interface, slight changes in the nanowire diameter). The dip corresponds to 3/4Δ*E* ≈ 127.5 meV diminished by the voltage drop at the resistive load (*R*_load_) accounting for the shell and core sections connecting the InAs-GaSb interface to contacts 1 and 2, respectively. Actually, with reference to Figure 1a, the current flows from the right electrode on the GaSb shell to the left electrode on the InAs core, i.e., it flows radially across the GaSb shell, then across the GaSb/InAs junction, then axially along the InAs core. In this frame, *R*_load_ accounts for the resistance of the semiconductor sections in series with the broken gap junction. Contact resistances not exceeding a few kOhm also contribute to *R*_load_. Device-dependent *R*_load_ in the range 10–100 kOhm is typically observed in our devices.

Moreover, the occurrence of a finite (nonzero) current minimum implies the presence of a parallel conduction path, i.e., a resistive leak (*R*_leak_). The two dissipative elements *R*_load_ and *R*_leak_ are combined with an Esaki diode (representing the broken-gap junction) in the circuital representation of the device discussed in the Supporting Information and are taken into account in our model of the device conductance. At a large magnetic field, e.g., *B* = 20 T, the experimental curves *I*_DS_ (*V*_DS_) and d*I*/d*V* (*V*_DS_) show two clearly resolved NDR features, as reported in Figure 2d,f. Consistent with the picture illustrated in Figure 1d,e, we attribute the doublet to the Zeeman-driven splitting of the band-edges (*E*_v_ and *E*_c_). The reported data were measured with *B* directed along the radius of the CS-NW (configuration *B*_radial_, see Figure 1a). However, analogous separations are observed when an axial component of *B* is introduced by tilting the sample (configuration *B*_tilted_). The experimental configurations are constrained by the high-field measurement setup, which does not allow for a full axial alignment of the field.

In Figure 3a,b we show two gray scale-color maps of the differential conductance of the device as a function of the magnetic field and the source-drain voltage for the two orientations of the magnetic field described above, namely, radial and tilted. At sufficiently large fields, from about 10 T up to 30 T, we can resolve and follow the evolution of two spin-split components. The limiting factor for the observation of the doublet at lower fields likely resides in the quality and homogeneity of the InAs-GaSb interface, which unavoidably introduces a broadening of the NDR features due to the spatially averaging nature of the electrical transport measurements across the interface. On the one hand, the separation between the positions of the two NDR features increase linearly with the magnetic field for both radial and tilted configuration of the field, suggesting a Zeeman-based origin of this phenomenology together with a linear evolution of the band-edges. On the other hand, the NDR features exhibit a dispersion toward large positive bias. To rationalize this feature, one needs to consider the contribution to the *I*_DS_ (*V*_DS_) curves determined by different sections of the NW involved in the transport, that we quantify by the two parameters *R*_load_ and *R*_leak_ defined previously. The electrical resistance ensuing from these circuital elements is indeed expected to display a marked *B*-dependence, with an overall quadratic behavior and the possible occurrence of sizable oscillations, as reported for instance in InAs NWs.^23,24^ In these nanostructures, large positive magnetoresistance and conductance modulations are manifestation of the magnetic field dependence of the 1D conducting states as well as the spin and orbital degeneracy, mediated by the *B*-dependence of the Fermi level. In our case, the field evolution of our device is quite accurately reproduced by incorporating both the evolution of the band alignment as a function of the magnetic field and the magnetoresistance of the passive elements. Accordingly, in our model the total current is a sum of two components: the tunneling current through the broken gap at the C–S interface, limited by *R*_load_, and the leakage current through the resistance *R*_leak_ connected in parallel.

**Figure 3:**
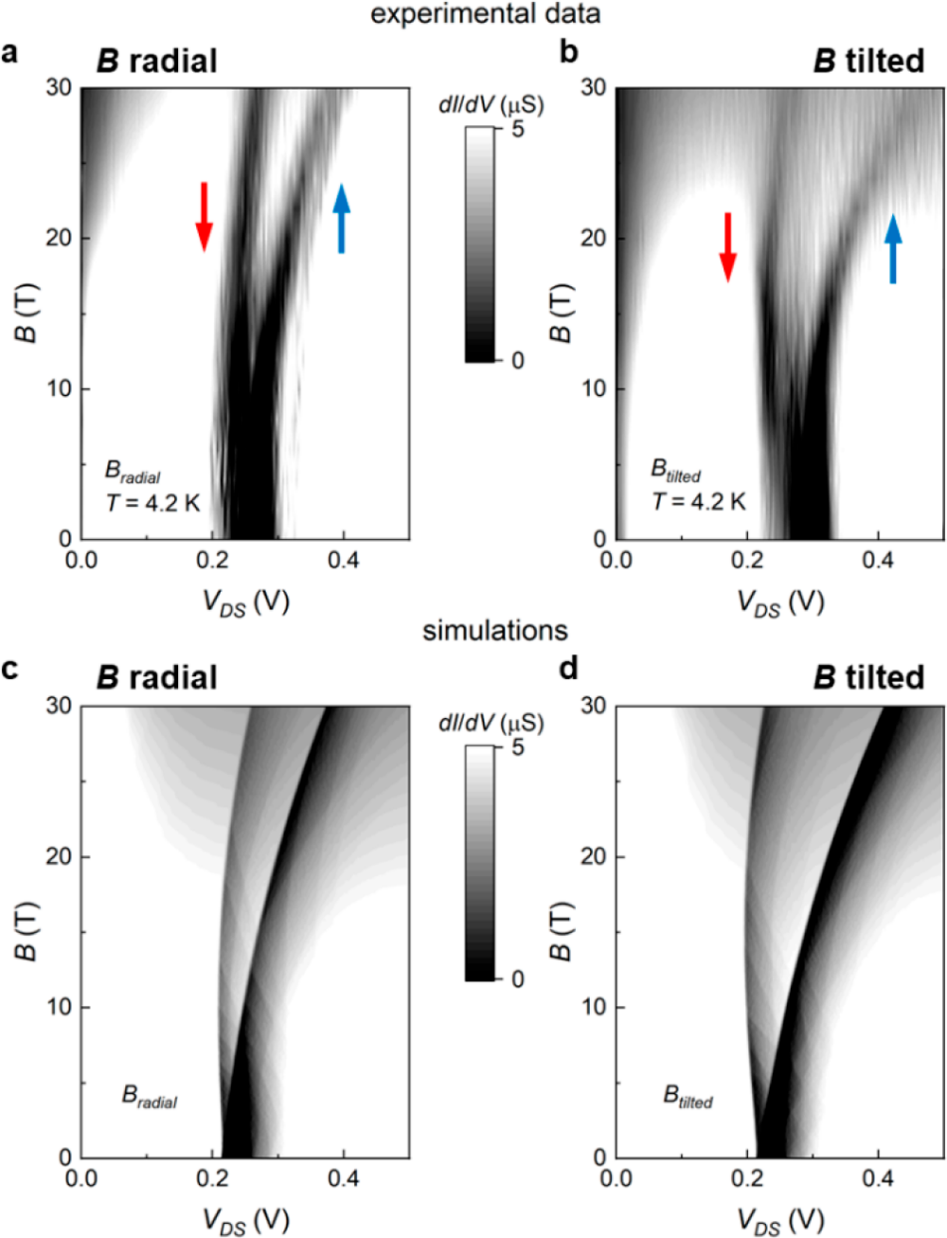
Color map of d*I*/d*V* as a function of *V*_DS_ and *B*, measured at 4.2 K, with magnetic field applied radial (a) or tilted (b) respect to the NW axis. (c, d) Simulated d*I*/d*V* maps, corresponding to the experimental data reported in (a) and (b). The low conductance region at low *V*_DS_ in (a) and (b) corresponds to a sublinear current voltage characteristic, broadened by the *B* field.

At low bias, the tunneling component is obtained from the Landauer formula, based on the electronic states evaluated from the envelope function equation, which is solved numerically for the prismatic NW geometry (full details are reported in Supporting Information, Section 2), including a magnetic field in the appropriate direction. On this basis, we produce simulated maps of the differential conductance, as the ones presented in Figure 3c,d, showing a dispersion in accordance to the experimental data. In the simulations, the splitting Δ*V*(*B*) is used as a fitting parameter, which in turns determines the difference in the effective *g*-factors between holes (in GaSb) and electrons (in InAs), Δ*g*. By this mean, we found that Δ*g* largely exceeds the value predicted by considering the bulk band structure parameters for the two materials, remarkably reaching *g* ∼ 66 and *g* ∼ 106 for the radial and tilted configurations, respectively. Notably, very large and anisotropic *g*-factors can be expected just on the base of a simple empirical analysis of the as-measured magneto-conductance reported in Figure 3a,b. In fact, if Δ*V*_DS_ is the voltage separation between the two spin-polarized branches of the NDR feature at a given magnetic field *B*, then *r*·*e*·Δ*V*_DS_ = *g*μ_B_*B*, where *r* = Δ*E*/e·Δ*V*_DS_ converts *V*_DS_ into an energy bias at the InAs/GaSb interface. This ratio can be extracted from the voltage position of the conductance minimum at *B* = 0, which correspond to a chemical potential aligned at approximately 3/4 of the broken gap, providing *r* = 127.5 meV/280 meV ≈ 0.46. Ultimately, at *B* = 30 T, the 115 mV separation between spin up and down NDR branches extracted from Figure 3a yields *g*_radial_ = 30.5, while the 200 mV split extracted from Figure 3b yields *g*_tilted_ = 53.

Overall, we carried out transport experiments in more than 10 nanodevices fabricated as depicted in Figure 1, and we observed transport features consistent with a NDR splitting in magnetic field in all devices used for high-field magneto transport measurements (Supporting Information, Section 3).

Finally, we note that, by showing the differential conductivity extracted from our data sets, we highlight the fine structure of the NDR, emphasizing the visibility of the NDR split due to the magnetic field. Regarding the *B*-dependence of the current, a strong magnetoresistance increase with *B* was trivially observed.

Figure 4a,b show the calculated energy dispersion of the lowest InAs and GaSb subbands at zero source-drain bias, at small or large magnetic fields for both field configurations. Given that Δ*g*, rather than the individual *g* factors, is the quantity of relevance for the NDR split, we take *g* = 0 for the GaSb shell (black lines) and *g* = Δ*g* for the InAs core (red and blue lines for the two spin components). For *k*_*z*_ = 0 states, at a given *B*, a finite *V*_DS_ drives the alignment between the lowest subband of each spin orientation in the two layers, determining the edge of the NDR region. By increasing further *V*_DS_, the two bands decouple, turning the broken gap configuration to a type-II gapped heterostructure. At finite *k*_*z*_, this condition is reached at smaller values of the bias. That is, from a different perspective, with increasing *V*_DS_, the tunneling current involves states with lower *k*_*z*_. However, one should take into account that the tunnelling current involves not only the lowest subbands, but a large number of them (e.g., up to 20 subbands at *B* = 2 T). In our simulations of Figure 3c,d, all relevant sub-bands are included (see Supporting Information, Section 2), and the values used for Δ*g* in the two magnetic field configurations are obtained from a linear fitting of the experimental Δ*V*(*B*) splitting of the two spin branches, as illustrated in Figure 4c. Moreover, while at low *B* field the sub-bands retain their almost-parabolic dispersion (Figure 4a,b at *B* = 4 T), at high fields sub-bands flatten for small wavevectors (Figure 4a,b at *B* = 20 T), indicating the formation of dispersionless Landau levels. The computed real-space distribution of such states, with and without an applied magnetic field, at *k*_*z*_ = 0 and *k*_*z*_ = 2 nm^–1^ is reported in Figure 4d. With a finite *B*, the localization pattern of the core electrons and of the shell holes loses the symmetry of the hexagonal cross-section and, at nonzero wave vector, the densities of the two carriers are pushed on a side of the hexagon. Note that here the sign of the wave vector represents the direction of the current along the NW. As a consequence, the two carriers with opposite charge are deflected in the same direction by the Lorentz force.

**Figure 4:**
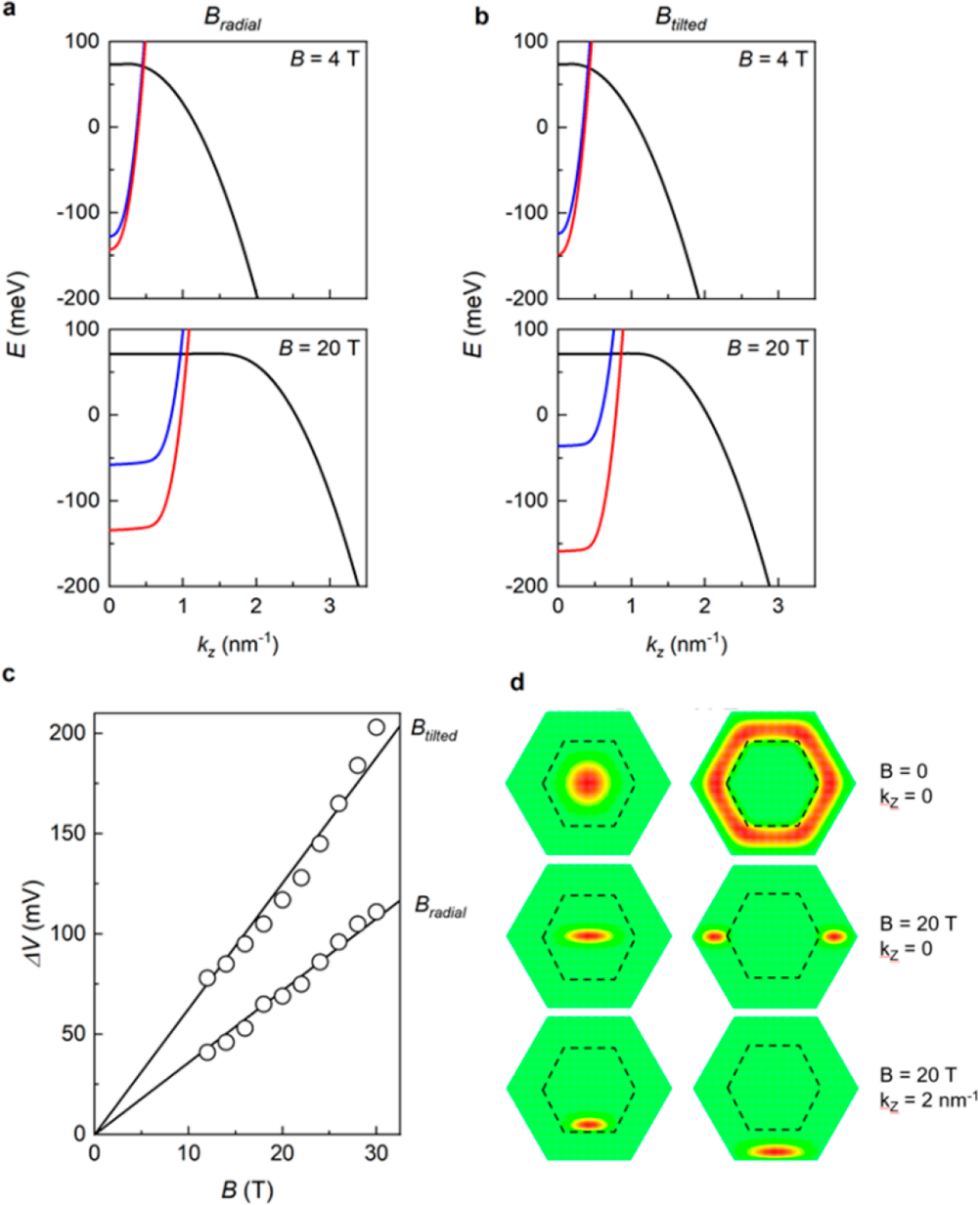
(a, b) Lowest sub-bands for the GaSb (black, for both spin up and down) and the InAs (red, spin up; blue, spin down) layers at different magnetic field intensities and configurations, indicated by the labels. (c) Difference of the source-drain bias voltages of the two spin-resolved NDR regions vs magnetic field, for tilted and radial configurations. Bullets correspond to the experimental data while solid lines are the numerical fitting used in the simulations. (d) Real-space distributions of the ground electronic wave function at zero and strong magnetic field and at zero and finite wave vector (see the labels on the right). Left (right) column shows the core (shell) electrons (holes) and the dashed hexagon indicated the InAs-GaSb interface.

NDR in C–S NWs was reported up to room temperature at zero magnetic field in ref (14). Given the large energy scales estimated from the NDR splitting at high magnetic fields, in principle such splitting could be relatively robust to thermal excitation. Figure 5a shows the temperature-dependent conductance measured in one of our devices at *B* = 20 T and, surprisingly, the NDR peak at the largest energy is rapidly suppressed as *T* increases. This unexpected experimental finding can be rationalized by taking into account a thermally activated transport between the valence bands of the two materials, which is expected at sufficiently high voltage, when the two bands align. We incorporate such a contribution in our model by introducing a current diode whose saturation current and ideality factor (see Supporting Information) are fitted to the experiments with a genetic algorithm, a stochastic global optimization algorithm (^25^ and the optimize library from the *scipy* python package), together with *R*_load_ and *R*_leak_. Figure 5b reports simulated temperature-dependent curves generated under the same conditions used experimentally. The simulations reproduce the suppression of the upper peak at the observed temperature. Superimposed to the split NDR temperature dependence highlighted in Figure 5, a strong resistance decrease with increasing *T* was trivially observed. This is also responsible for the shift of the NDR dip toward lower voltages.

**Figure 5:**
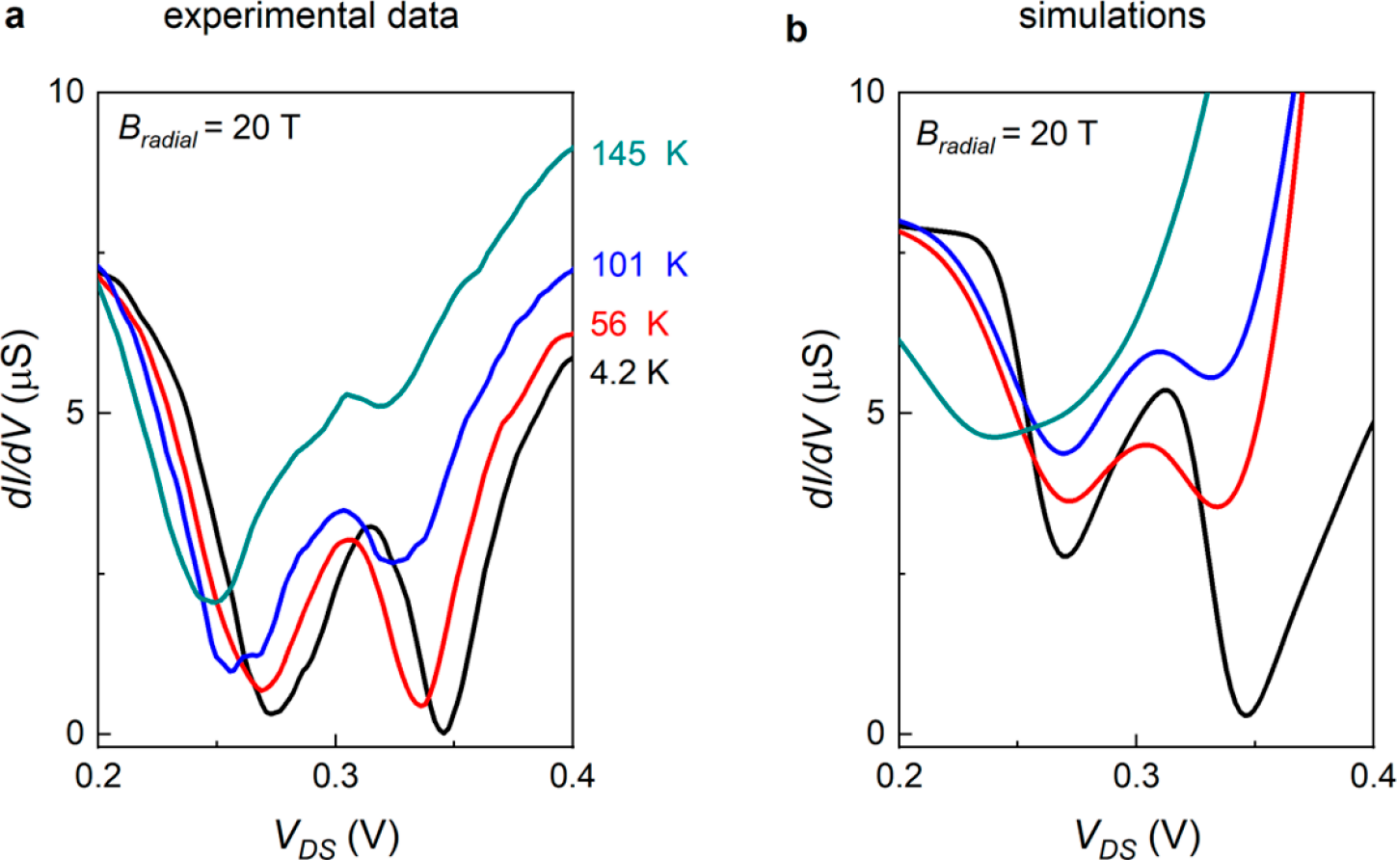
(a) Differential conductance for increasing temperatures, at constant magnetic field (20 T). (b) Simulated d*I*/d*V* curves, under the same conditions as in (a).

While our model reproduces quite well the NDR evolution with *B* and its temperature dependence, confirming the assignment of the NDR peaks and allowing to extract a very large *g*-factor, only a complete theoretical description involving full band structure calculation, accounting for spin–orbit coupling in the presence of a large magnetic field, could provide a clue to such large values of *g*. However, it has been suggested that large spin–orbit constants can be induced, and tuned, by nonsymmetric dielectric configurations,^26,27^ while magnetic and electric fields may also combine and contribute to the *g* anisotropy^28^ reported in our experiments and also highlighted in other experimental works.^29,30^ Moreover, a giant *g*-factor has been predicted for higher sub-bands in an axial field configuration due to the large angular momentum of the quantum states along preferential direction in NW-based devices,^13^ and this may contribute to the large anisotropy observed in our device when the field is tilted along the NW axis. Finally, in the broken gap alignment, hybridization between conduction and valence states of the two layers may take place, thus contributing to the giant *g*-factor.^31^ In conclusion, we have demonstrated the possibility to magnetically induce and electrically address the spin-polarization of carriers in ambipolar core–shell nanowires. On the one hand, this outcome can be of relevance for the investigation of fundamental phenomena such as Coulomb drag in low dimensional nanostructures as well as the occurrence of novel topological phases of matter due to electron–hole interactions. On the other hand, in the presence of temperature gradients, it may open the way to innovative approaches to nanoscale thermoelectrics. Finally, the C–S NW system may realize a quasi-1D spin filter of relevance for spintronic technologies.

## Supporting Information Available

Description of the 8-band *k⃗*·*p⃗* calculations of the spin–orbital states in the section of the core–shell nanowire; Modeling of the quantum transport through the broken-gap Esaki junction based on the equivalent electronic circuit; Additional data sets measured at low *B* in a second device (PDF)
