# Supplementary material for: Spin-Resolved Magneto-Tunneling and Giant Anisotropic g-Factor in Broken Gap InAs-GaSb Core–Shell Nanowires
Source: Nano Lett. 2024 Jan 8;24(3):790–6. doi: 10.1021/acs.nanolett.3c02559 (PMC10811674; doi:10.1021/acs.nanolett.3c02559)
Supplement: Supplementary file 1 — nl3c02559_si_001.pdf [file nl3c02559_si_001.pdf]

SUPPORTING INFORMATION

Spin-resolved magneto-tunneling and giant anisotropic *g*-factor in broken gap InAs-GaSb core-shell nanowires

Vito Clericò[1], Pawel Wójcik[2], Andrea Vezzosi[3], Mirko Rocci[4], Valeria Demontis[4], Valentina Zannier[4], Álvaro Díaz-Fernández [5], Elena Díaz[5], Vittorio Bellani[1,6], Francisco Domínguez-Adame[5], Enrique Diez[1], Lucia Sorba[4], Andrea Bertoni[7], Guido Goldoni[3] and Francesco Rossella[3,*]

[1] *Nanolab-Nanotechnology Group, Departamento de Física Fundamental, Universidad de Salamanca, Plaza de la Merced, s/n. 37008-Salamanca, Spain*

[2] *AGH University of Krakow, Faculty of Physics and Applied Computer Science, Al. Mickiewicza 30, 30-059 Krakow, Poland*

[3] *Dipartimento di Scienze Fisiche, Informatiche e Matematiche, Università di Modena e Reggio Emilia, Via Campi 213/a, I-41125 Modena, Italy*

[4] *NEST, Scuola Normale Superiore e Istituto di Nanoscienze-CNR, Piazza san Silvestro 12, I-56127 Pisa, Italy*

[5] *GISC, Departamento de Física de Materiales, Universidad Complutense de Madrid, Avenida Complutense, s/n, Ciudad Universitaria, 28040 Madrid, Spain*

[6] *Dipartimento di Fisica, Università di Pavia, Via Agostino Bassi, 6, 27100 Pavia, Italy*

[7] *S3, Istituto Nanoscienze-CNR, Via Campi 213/a, I-41125 Modena, Italy*

* *Corresponding author. E-mail: francesco.rossella@unimore.it*

SI – 1: $\vec{k} \cdot \vec{p}$ BAND CALCULATIONS

We have performed $\vec{k} \cdot \vec{p}$ calculations with a 8x8 Kane Hamiltonian, using a real-space representation within the Finite Element Method supplemented by a symmetry-adapted (SAFE) representation [[1]] to avoid spurious solutions. We use Burt-Foreman boundary conditions between the InAs core (60 nm edge-to-edge) and the Gasb shell (30 nm edge-to-edge), both

segments being nominally undoped, on a inhomogeneous grid of ~3000 elements. Band parameters are taken from [2]. The quantization axis of the total angular momentum is taken along the [111] free axis direction (hence the light-hole states have larger transverse masses and smaller confinement energies) and subband energies are calculated as a function of the wavevector k_z along the free nanowire axis. Details of the numerical implementation are given in [3]. In Fig. S1 we plot the subband energies at $k_z = 0$ in the range between the InAs conduction band edge, E_c, and the GaSb valence band edge, E_v, i.e. where the bulk band structures of the two materials overlap in energy. For each state we report the conduction electron character (EL, blue dots), the light-hole character (LH, green dots) and heavy-hole character (HH, orange dots), defined as the fraction of the spinor with a corresponding orbital character, as in Ref. [3], normalized to unity. Calculations show clearly that the nano-device is deeply in the band-inverted regime, with many states between the two band edges. All states have a strongly hybridized LH/HH character, arising from the GaSb shell, and EL character, arising from the InAs core, the overall dominating character being LH. However, some states have a stronger EL character (and a correspondingly smaller LH character) which is reminiscent of the conduction subband edges which, at large k_z (not shown here), will eventually leave the region of the overlapping gap as almost pure conduction state subbands. Note that conduction-like states have an increasing EL character as the energy increases, and above E_v, they show dominating EL character. Due to the large density of state, no hybridization gap opens at finite k_z [4], and the material is confirmed to have a metallic character.

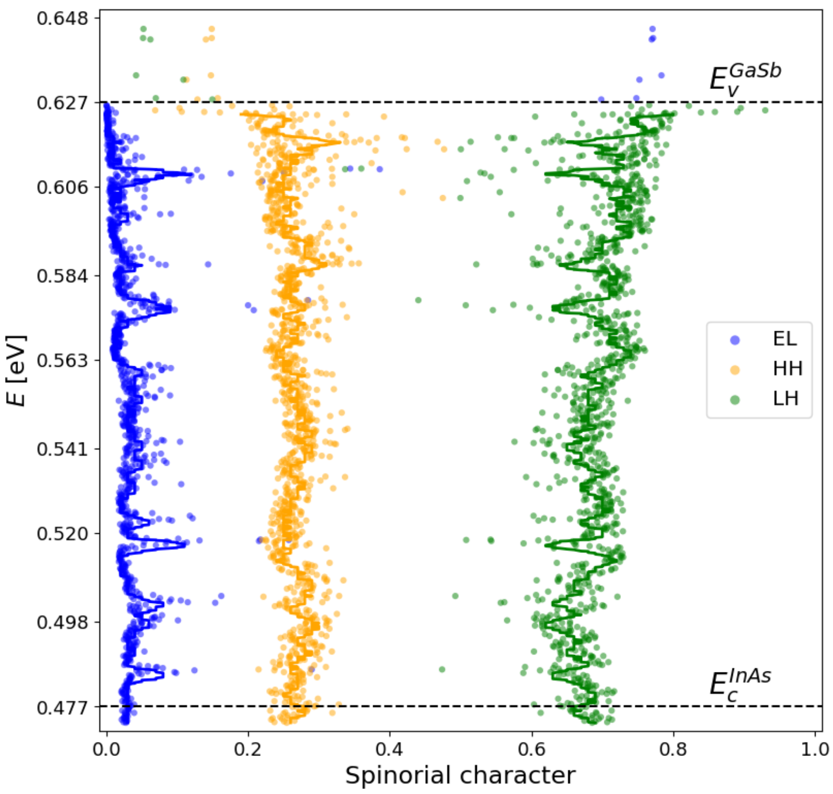

Figure S1 Subband energies and corresponding conduction (blue dots), heavy-hole (orange dots) and light-hole (green dots) spinorial character. For clarity, we show as full lines the moving averages of the corresponding data sets. The reference energy is the InAs bulk valence band edge, E_V^{InAs}.

SI – 2: MODELING OF TRANSPORT

The current from the source to the drain electrode, in the experimental setup presented in Fig. 1b, is modeled as (see Fig. S2a) the sum of (i) the current across the core/shell interface, showing an NDR feature analogous to an Esaki diode, limited by a series resistance R_{load} formed by the material segments connecting the InAs-GaSb interface to the source and drain contacts, and (ii) the leakage current which includes all unknown alternative current paths, limited by the effective resistance R_{leak} connected in parallel. The former contribution, in turn, is composed of a low-bias tunneling current across the inverted interface I_{tun} (Fig. S2b) and a high-bias diode current I_{diode} corresponding to the thermally activated transport processes between the valence bands of the two materials.

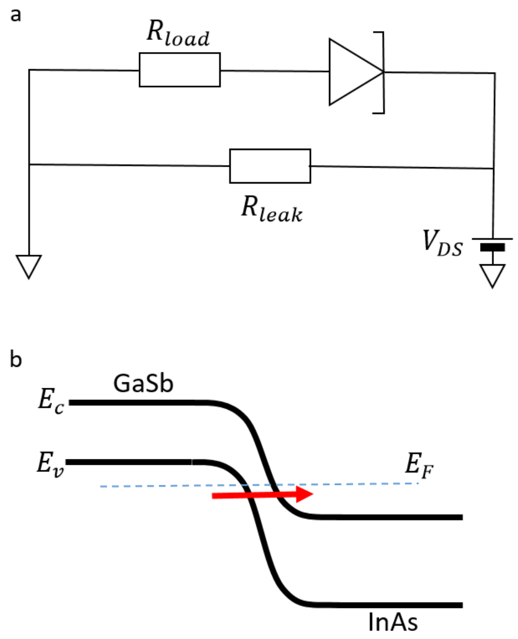

Figure S2. *(a) Sketch of electronic circuit used for the transport modelling. (b) Schematic illustration of the band diagram at the GaSb/InAs interface with the inversion of bands.*

Therefore, the total current I_{DS} is given by

$$I_{DS} = I_{tun} + I_{diode} + \frac{V_{DS}}{R_{leak}}$$

Below we discuss the calculation of each term.

The diode current I_{diode} - The diode component is assumed in the common form [5]

$$I_{diode} = I_s exp\left[\frac{eV_{DS}}{\eta k_B T} - 1\right]$$

where I_s is the saturation current and η is the ideality factor, which are fitted to the experimental curves.

The tunneling current I_{tun} – Due to band inversion at the InAs-GaSb interface, the tunneling current I_{tun} is equivalent to the transport through the reversed polarized Esaki diode. Electrons from the valance band of the GaSb tunnel towards the strongly spin-split empty states in the InAs core, whereas the transport through the interface is determined by the relative energy of the conduction and valance bands in the core and shell, as displayed in Fig. S1(b). If the energy of the conduction band edge in the InAs core is higher than the energy of the valance band edge in the GaSb shell the tunneling transport is completely blocked.

Within the two-current model [6] the tunneling component I_{tun} thorough the InAs-GaSb interface is a sum of spin current components
$I_{tun} = I_{tun}^{\uparrow} + I_{tun}^{\downarrow}$ given by the Landauer formula

$$I_{tun}^{\sigma} = \sum_n e \int dk_z v_n^{\sigma} T_n^{\sigma} f_{FD}\left(k_z, E_F + \frac{V_{DS}}{2}\right)\left(1 - f_{FD}\left(k_z, E_F - \frac{V_{DS}}{2}\right)\right)$$

where the summation is carried over all electronic states labeled by the subband index n, k_z is the wave vector along the nanowire axis, $v_n^{\sigma} = (\frac{1}{\hbar})\frac{\partial E_n^{\sigma}(k_z)}{\partial k_z}$ is the group velocity, E_F is the Fermi

energy (we assume E_F in the middle of the broken gap), f_{FD} is the Fermi-Dirac distribution function and T_n^σ is the transmission coefficient calculated from the WKB approximation, assuming the commonly used triangle shape potential at the interface $V(z) = E_g\left(1 - \frac{z}{W}\right)$, where E_g is the height of the barrier potential while W is the barrier characteristic length. According to the WKB approximation

$$T_n^\sigma(k_z) = e^{-2\alpha} = exp\left(-\frac{4W}{3}\sqrt{\frac{2mE_g}{\hbar^2}}\right) exp\left(\frac{2W}{E_g}\sqrt{\frac{2mE_g}{\hbar^2}}E_n(k_z)\right) = T_0 exp\left(\frac{E_n(k_z)}{E_0}\right)$$

where finally we treat T_0 and E_0 as fitting parameters.

The electronic structure $E_n^\sigma(k_z)$ of the C-S NW is determined by solving the eigenproblem of the Hamiltonian

$$\hat{H} = \frac{1}{2}(\hat{p} - eA(r))\frac{1}{m^*(r)}(\hat{p} - eA(r)) + E_{c(v)}(r) + \frac{1}{2}g\mu_B\sigma \cdot B$$

where $\hat{p}$ is the conjugate momentum operator, $A(r)$ is the magnetic vector potential, $m^*(r)$ is the effective mass, $E_{c(v)}$ is the conduction (valence) band edge, g is the gyromagnetic factor and $\sigma = (\sigma_x, \sigma_y, \sigma_z,)$ is the vector of Pauli matrices.

For a given magnetic field B, the envelope function equation is solved numerically for the hexagonal geometry on a triangular grid [7]. The maxima of the valence band in GaSb is set as the reference energy $E_v = 0$ assuming the energy band offset at the InAs-GaSb interface $E_{off} = 0.876$ eV. Our single band approach neglects the electron-electron interaction and the coupling between the conduction and valence band at the InAs-GaSb interface. The problem is solved separately for electrons in the InAs core ($m^* = 0.023\, m_0$) and holes in the GaSb shell ($m^* = 0.4\, m_0$). Following the experiment, we consider two configurations of the magnetic field

(a) radially oriented and (b) tilted by 45° with respect to the NW axis. The dispersion $E_n(k_z)$ of the lowest subbands calculated for both magnetic field orientations are presented in Fig. 4(a,b).

The fitting procedure is optimized based on the genetic algorithm from the SciPy library, using R_{leak}, $R_{load,}$ T_o and E_0 as the fitting parameters at each magnetic field. Best fit is obtained for the gyromagnetic factor $g = 66$ and $g = 106$ in the radial and tilted field configurations, respectively. If not state otherwise, calculations were performed at $T = 4.2$ with the diode current $I_{diode} = 0$.

In Fig. S3 we show the current-voltage characteristics $I_{DS}(V_{DS})$ for selected magnitude of the radial magnetic field. The full maps $\frac{dI_{DS}}{dV_{DS}(V_{DS},B)}$ are presented in Fig. 3 (e,f) in the main manuscript.

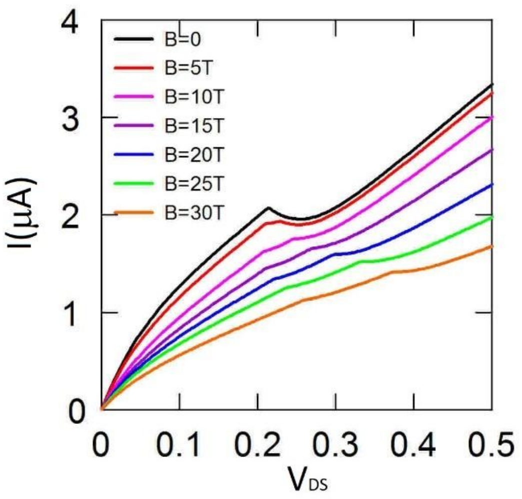

Figure S3. *The simulated current-voltage characteristics $I_{DS}(V_{DS})$ for selected magnitude of the radial magnetic field.*

When increasing temperature, the thermally transport processes activate giving raise to $I_{diode} \neq 0$. The inclusion of this current component could *inter alia* lead to the experimentally observed vanishing of the NDR for the higher temperature, see Fig. 5 (main manuscript). In our simulations we treat the saturation current I_s as the parameter assuming the ideality factor η=5 regardless of the temperature.

SI – 3: DOUBLE NDR FEATURES AT LOW B IN A SECOND DEVICE

Overall, magneto-transport experiments in different magnetic field ranges have been performed in several nanodevices nominally identical to the one shown in Fig.1 of the main text (device A), observing a double NDR feature even at low and intermediate magnetic field.

As an example, left panels of Figure S4 report the I-V curves measured in a second device (device B, not discussed in the main text) in radial magnetic field in the range 5-10 T with both positive and negative direction. Device current was measured sweeping the source-drain bias, V in forward and reverse directions in the range from -0.4 V to 0.4 V. This originates the hysteretic features observed in the characteristics I-Vs. For negative V, no NDR is observed, as expected. For positive V, a double NDR is observed around 0.3 V for all applied magnetic fields.

Right panel of Figure S4 report the colorplot of the differential conductance measured in a third device (device C, not discussed in the main text) in tilted magnetic field in the range 0-20 T, evidencing the onset of two Zeeman-splitted NDR branches.

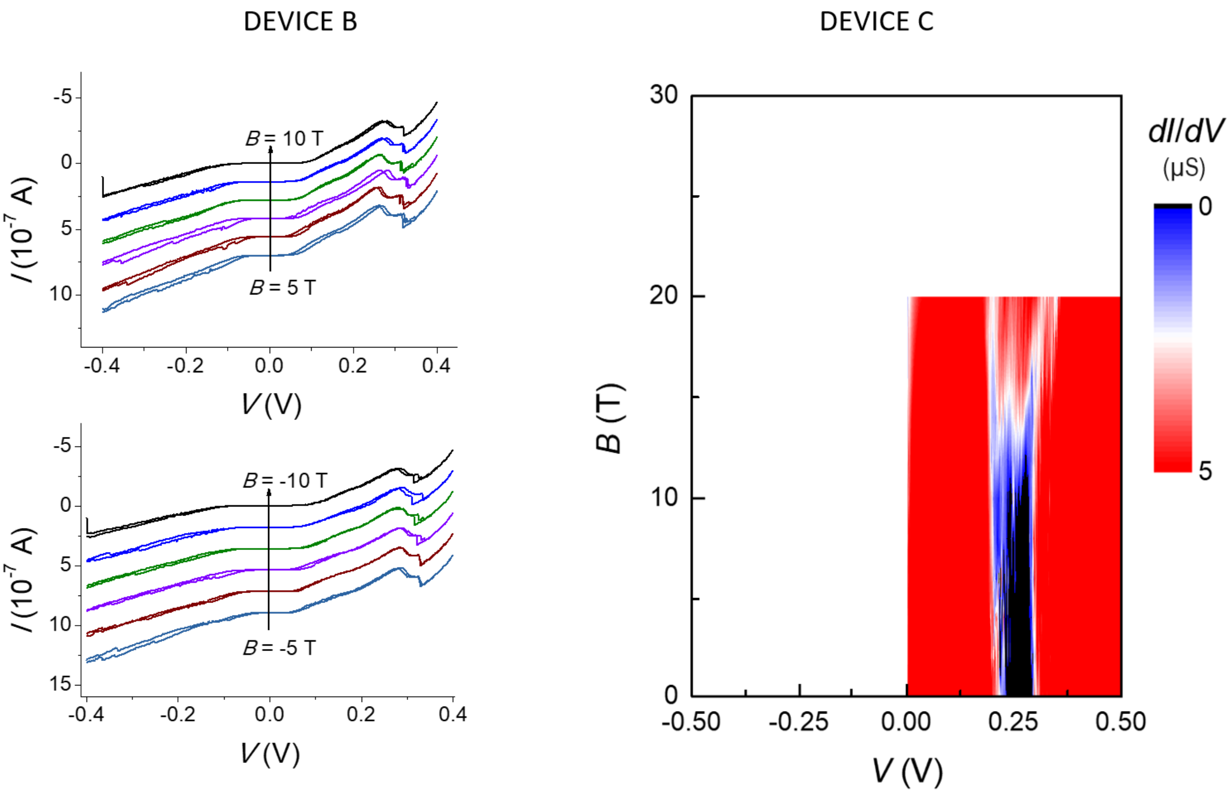

Figure S4. *I-V characteristics measured in device B in presence of a radial magnetic field applied with both positive (top-left) and negative (bottom-left) directions. Differential conductance measured in device C for positive source-drain bias, V and tilted magnetic field in the range 0-20 T (right).*

References:

[1] T. Eissfeller and P. Vogl, Real-space multiband envelope-function approach without spurious solutions, Phys. Rev. B 84, 195122 (2011)

[2] I. Vurgaftman, J. R. Meyer, and L. R. Ram-Mohan, Band parameters for III–V compound semiconductors and their alloys, J. App. Phys. 89, 5815 (2001)

[3] Vezzosi A., Bertoni A., and Goldoni G. Band structure of n- and p-doped core-shell nanowires. Phys. Rev. B 105, 245303 (2022)

[4] N. Luo, G.-Y. Huang, G. Liao, L.-H. Ye, and H. Q. Xu, Band-inverted gaps in InAs/GaSb and GaSb/InAs core-shell nanowires, Sci. Rep. 6, 38698 (2016)

[5] S. M. Sze, Kwok K. Ng, ”Physics of semiconductor devices”, John Wiley and Sons, Inc. Hoboken, New Jersey, 2007, ISBN-13:978-0-471-14323-9

[6] N.H.Mott, Proc. Roy. Soc. A 153, 699 (1936)

[7] M. Royo, A. Bertoni, and G. Goldoni, “Landau levels, edge states, and magnetoconductance in GaAs/AlGaAs core-shell nanowires” Phys. Rev. B 89, 155416 (2014)
